# Native Hawaiian and Pacific Islander Patients with Fibromyalgia: Insights into BMI, Pain, and Comorbidities in a Multiethnic Cohort

**DOI:** 10.21203/rs.3.rs-8497320/v1

**Published:** 2026-01-20

**Authors:** Matthew Kao, Andrew Mettias, Cameron Nishida, Bryce Hong, Ryan Nishi, Noa Brenner, Kyle Ishikawa, Natalia Gonzalez, Kore Liow, Enrique Carrazana

**Affiliations:** Hawaii Pacific Neuroscience; Hawaii Pacific Neuroscience; Hawaii Pacific Neuroscience; Hawaii Pacific Neuroscience; Hawaii Pacific Neuroscience; John A. Burns School of Medicine, University of Hawaii; John A. Burns School of Medicine, University of Hawaii; Hawaii Pacific Neuroscience; Hawaii Pacific Neuroscience; Hawaii Pacific Neuroscience

**Keywords:** Fibromyalgia, Native Hawaiians, Pacific Islanders, Body mass index, Health disparities, Biopsychosocial model

## Abstract

**Background:**

Native Hawaiian and Pacific Islanders (NHPI) are significantly underrepresented in fibromyalgia literature. This study aims to characterize the clinical presentation of fibromyalgia across ethnoracial groups to identify potential disparities in symptoms and comorbidities.

**Methods:**

A retrospective chart review was conducted at a single-center clinic involving 200 patients diagnosed with fibromyalgia. Patients were stratified by race: Native Hawaiian, Pacific Islander, Asian, and White. Clinical variables including Body Mass Index (BMI), pain severity, exercise frequency, and comorbidities were analyzed using ANOVA and Chi-Square tests with Bonferroni corrections.

**Results:**

The primary ethnoracial difference identified was BMI (p=0.0336). Mean BMI was highest in Pacific Islander and Native Hawaiian groups compared to White and Asian. While pain and depression did not differ significantly by race, higher BMI was significantly associated with increased pain severity (p=0.009), higher prevalence of diabetes (p=0.004) and hypertension (p=0.006), and lower exercise frequency (p=0.045). Patients with high pain scores also showed higher depression rates compared to those with low scores (p=0.024).

**Conclusions:**

BMI may be a key pathway for elucidating ethnoracial clinical differences in fibromyalgia presentation, with higher BMI being linked to increased pain severity, higher depression rates, and a greater burden of cardiometabolic comorbidities. While analyses by race alone did not display these relationships, NHPI patients presented with disproportionately higher BMIs. This suggests that BMI may act as a key mediator for ethnoracial health disparities, highlighting the importance of culturally informed and multidisciplinary management that integrates pain care, mental health, and metabolic risk reduction.

## Background

Fibromyalgia is a complex and often debilitating chronic pain disorder characterized by widespread musculoskeletal pain, fatigue, sleep disturbances, cognitive dysfunction, and mood changes. The 2012 National Health Interview Survey found that fibromyalgia affects an estimated 1.75% of the U.S. adult population, representing about 3.94 million people at the time [1]. Furthermore, another study found the global prevalence of fibromyalgia in the general population at 1.78% (95% CI: 1.65-1.92), predominantly impacting women with a higher rate of 3.98% (95% CI: 2.80-5.20) compared to the general global population [2].

The mechanisms underlying fibromyalgia are not fully understood, and outcomes are highly variable. This variability highlights the importance of considering biopsychosocial factors in understanding and managing the condition [3]. Despite its prevalence, much of the foundational research and nearly all clinical trials for fibromyalgia have been conducted in predominantly non-diverse patient populations, with approximately 90% of patients identifying as White, with no significant change in this trend over the past 22 years [4]. This significant lack of diversity creates a critical knowledge gap and overlooks potential ethnoracial differences among disease presentation and treatment.

Hawaii’s unique landscape as a minority-majority state provides a valuable opportunity to address this gap in literature. With the state’s prevalent populations of Native Hawaiian and Pacific Islander (NHPI) and Asian patients being a few of those largely underrepresented groups, this provides an opportunity to better characterize how clinical presentation of fibromyalgia may differ across ethnoracial groups. Elucidating this potential difference could prove invaluable in terms of disease recognition and tailoring of management strategies. Further, there is a known growing trend of NHPI patients in Hawaii moving away to the continental United States for a variety of reasons [5]. Knowing this, being aware of potential ethnoracial differences in the presentation and clinical approach to this patient population becomes relevant for clinics both within and outside of Hawaii. Thus, the primary objective of this study is to characterize and compare the clinical presentation of fibromyalgia across ethnoracial groups within our diverse population of Hawaii, focusing on demographic, clinical, and biopsychosocial variables.

## Methods

This study was conducted at Hawaii Pacific Neuroscience (HPN), a single-center outpatient neurology clinic. This study was approved by the University of Hawaii Institutional Review Board (IRB #2020-01010). A retrospective chart review of patients was conducted based on the following criteria: 1) were diagnosed with fibromyalgia, either as a primary or secondary diagnosis, 2) had complete ethnoracial data on record, and 3) were seen at HPN between 2010 and 2025. Fibromyalgia diagnoses were made by board-certified neurologists using standard clinical criteria consistent with American College of Rheumatology guidelines (2016). Formal Widespread Pain Index (WPI) and Symptom Severity Scale (SSS) scores were not uniformly documented due to the retrospective nature of the study.

Data was extracted from the clinic’s electronic health records. The following variables were collected: Age at diagnosis, sex, race/ethnicity (Native Hawaiian, Pacific Islander, Asian, and White), and Body Mass Index (BMI) recorded as a continuous variable. Pain severity was captured using a numeric rating scale (0-10) documented at the time of diagnosis, along with an updated value respective to each follow-up. The presence of concurrent clinical diagnoses and comorbidities were recorded as a binary (yes/no) variable. Exercise frequency was collected as self-reported days of physical activity per week.

Statistical analyses were conducted in Python (v3.11.2) using the Pandas library (v1.5.3). Group comparisons used ANOVA or Kruskal-Wallis tests for continuous data and Chi-square or Fisher's Exact tests for categorical data. A Bonferroni correction was applied for multiple comparisons, with significance set at an adjusted p-value < 0.05. Subgroup analyses were also performed by stratifying the cohort by BMI category (Normal ≤25, Overweight 26-29, Obese ≥30) and by a split median pain score (Low <7 vs High ≥7).

## Results

### Demographics and Baseline Characteristics:

The study cohort consisted of 200 patients diagnosed with fibromyalgia. The majority of patients were female (n = 160, 80%). The cohort was racially diverse, consisting of Native Hawaiian (n = 55, 27.5%), Pacific Islander (n = 40, 20%), Asian (n = 35, 17.5%), and White (n = 70, 35%) patients.

### Clinical Characteristics by Ethnoracial Group:

A comparison of key clinical variables across the four self-reported ethnoracial groups revealed a statistically significant difference in BMI after Bonferroni correction (p = 0.0336). The mean BMI was highest in the Pacific Islander group (30.1 ± 6.0), followed by the Native Hawaiian group (29.4 ± 5.0), then the White group (27.1 ± 5.4), and was lowest in the Asian group (24.8 ± 4.5). No other variables showed statistically significant differences between the groups after correction, see Table 1.

The mean age at diagnosis was 52.6 ± 15.3 years for Native Hawaiians, 49.7 ± 14.6 for Pacific Islanders, 47.8 ± 13.9 for Asians, and 54.1 ± 16.1 for Whites (adjusted p = 1.0). Mean pain scale scores were 6.5 ± 2.4 for Native Hawaiians, 7.0 ± 2.1 for Pacific Islanders, 5.3 ± 2.2 for Asians, and 6.8 ± 2.6 for Whites (adjusted p = 1.0). The prevalence of depression was (n = 24, 44%) in Native Hawaiians, (n = 20, 50%) in Pacific Islanders, (n = 9, 26%) in Asians, and (n = 28, 40%) in Whites (adjusted p = 1.0). While a trend was observed, the difference in mean exercise days per week did not reach statistical significance (Native Hawaiian 2.1 ± 1.3, Pacific Islander 2.3 ± 1.5, Asian 3.5 ± 1.6, White 3.2 ± 1.7; adjusted p = 0.064).

### Association of BMI and Clinical Outcomes:

When patients were stratified into three BMI categories (Normal ≤25, Overweight 26-29, Obese ≥30), several clinical variables showed a significant association with BMI after Bonferroni adjustment, see Table 2. Higher BMI was significantly associated with older age, with mean age being 48.5 ± 15.2 for the Normal group, 50.7 ± 14.9 for the Overweight group, and 54.9 ± 13.3 for the Obese group (p = 0.018). Higher BMI was also associated with higher pain scale scores, with mean scores for the Normal group being 5.5 ± 2.0, Overweight group 6.9 ± 2.3, and Obese group 7.4 ± 2.5 (p = 0.009). The prevalence of diabetes was significantly higher with increasing BMI as rates in the Normal group were (n = 10, 13%), Overweight (n = 14, 20%), and Obese (n = 26, 29%) (p = 0.004). Similarly, the prevalence of hypertension was significantly higher with increasing BMI as rates for the Normal group were (n = 13, 16%), Overweight (n = 16, 23%), and Obese (n = 28, 31%) (p = 0.006). Lastly, when comparing exercise frequency between Normal (3.3 ± 1.5 days per week), Overweight (2.9 ± 1.3 days per week), and Obese (2.2 ± 1.2 days per week) patients, higher BMI was associated with lower exercise frequency (p = 0.045).

### Association of Pain Severity and Clinical Outcomes:

The patient cohort was split based on their median pain score into a "Low Pain" group (<7, n = 104) and a "High Pain" group (≥7, n = 96), see Table 3. After Bonferroni correction, patients in the High Pain group had a significantly lower exercise frequency (mean 2.5 ± 1.5 days per week) compared to the Low Pain group (mean 3.2 ± 1.5 days per week) (p = 0.048). The prevalence of depression in the High Pain group was (n = 52, 54%) compared to the Low Pain group (n = 35, 34%), with depression rates being significantly greater in the High Pain group (p = 0.024). The mean BMI in the High Pain group (29.0 ± 5.7) was higher than in the Low Pain group (26.8 ± 5.3), but this association did not reach statistical significance after correction (adjusted p = 0.11).

### Analysis by Gender:

A comparative analysis between male (n = 40, 20%) and female (n = 160, 80%) patients revealed no statistically significant differences for any of the studied variables after Bonferroni correction. The distribution of race/ethnicity, BMI, age, pain scale, and the prevalence of comorbidities were comparable between genders.

## Discussion

In this multiethnic cohort of patients with fibromyalgia, we identified BMI as the primary ethnoracial difference, with NHPI patients presenting with significantly higher BMIs in comparison to their white and Asian counterparts. Interestingly, NHPI patients also displayed lower, but statistically insignificant, rates of reported exercise frequency. These findings align with existing literature showing elevated BMI in NHPI populations [6,7]. However, these findings may also suggest that BMI exists as a pathway through which ethnoracial differences manifest in patients with fibromyalgia. Thus, while BMI may not be a race-specific risk factor for fibromyalgia itself, it may help to explain how other comorbidities for fibromyalgia appear to differ across racial groups.

Specifically, when comorbidities were examined by BMI category, several clinically meaningful patterns emerged. Higher BMI was associated with increased subjective reports of pain, greater prevalence of diabetes and hypertension, increased rates of depression, and lower exercise frequency. These relationships reflect established trends which emphasize the interconnected nature of metabolic factors, physical activity, psychological symptoms, and pain perception; one study found that individuals with higher BMI reported substantially higher rates of daily pain with a graded increase across BMI categories [8], while another study found that higher BMI increases the risk of depression, especially in women [9]. Given this previously established data in combination with the disproportionate distribution of BMI across ethnoracial groups in our cohort, these BMI-stratified findings suggest that BMI may help explain how certain comorbidities appear more frequently in NHPI patients despite initial race-based comparisons not revealing significant differences. This could suggest a potential clinical pathway where the interplay between high metabolic burden and physical inactivity impacts patients’ pain and symptoms of depression in fibromyalgia. Essentially, BMI may act as a modulating factor through which underlying health disparities become visible with our fibromyalgia patients.

Importantly, these findings suggest that differences in fibromyalgia symptom burden across ethnoracial groups may be better understood through contextual and cardiometabolic factors rather than intrinsic differences in fibromyalgia phenotype. In our cohort, race itself was not independently associated with pain severity, depression, or other core fibromyalgia features after correction for multiple comparisons. Instead, higher BMI appeared to account for much of the variation in clinical presentation. This pattern supports a model in which fibromyalgia symptom expression is modulated by overall metabolic and psychosocial burden, rather than reflecting biologically distinct disease subtypes across racial groups.

Pain-related findings further reinforced the biopsychosocial nature of fibromyalgia. Patients in our cohort reported a median pain score of 7, consistent with prior studies. This score reflects the high burden of pain typically reported in fibromyalgia cohorts, where median pain scores on standard scales such as the Numeric Rating Scale frequently fall within the 5-8 range [10]. Those who reported scores above the median demonstrated statistically significant higher rates of depression and lower exercise frequency. Additionally, while not statistically significant, those with pain scores above the median also followed a trend towards elevated BMI levels. Thus, this variety of relationships underscores the complex, multidirectional interactions that exist between psychological, behavioral, and biological contributors in fibromyalgia.

These findings are particularly notable when paralleling them with NHPI individuals’ specific exercise habits. Despite an emphasis on “cultural” exercise such as outrigger paddling and hula dance (one study found that approximately 48.8% of Native Hawaiians have engaged in hula and 41.5% in outrigger canoe paddling, while among Other Pacific Islanders, 35.3% have engaged in hula and 31.1% in paddling [11]), overall physical activity levels among NHPI patients falls short of recommended guidelines with nearly 80% classified as insufficiently active in general population studies [12]. Noting this at baseline, our fibromyalgia patients may be even further limited by mentioned factors such as chronic pain and depression, further exemplifying the complexity of fibromyalgia and the multiple avenues for treatment. Namely, by understanding the emphasis that our patient population places on culture, it may be beneficial to address BMI and symptom management through encouragement of these sorts of traditional “cultural” exercises. Thus, taken together, the disparities in fibromyalgia presentation noted in our study reflect the interplay of biopsychosocial factors that must be considered for proper clinical care.

While our study provides valuable insights as one of the first studies exploring the clinical presentation of fibromyalgia among NHPI patients, several limitations should be considered. The sample size within each ethnoracial subgroup was relatively modest, which may limit the generalizability of our findings and the ability to detect subtle differences. Given the exploratory nature of this study, analyses were intended to be hypotheses generating. Multivariable regression was not performed because the limited subgroup sizes could have led to model overfitting and unstable parameter estimates. Also, by commenting on Native Hawaiian and Pacific Islander patients as one collective NHPI group, there is a potential for overlooking differences that may exist between these two patient populations. Additionally, the retrospective design and reliance on self-reported data, such as exercise frequency, introduce potential biases and limit causal inferences. We also acknowledge that cultural factors unique to NHPI communities that may influence health behaviors and symptom reporting were not fully explored in this study. Furthermore, other important confounders, including socioeconomic status and access to healthcare, were not addressed but likely play a role in observed disparities. Future research with larger, prospective cohorts that integrate cultural, social, and economic variables will be needed to further our understanding and improve care for this underserved population.

## Conclusions

Effective management of fibromyalgia requires a multifaceted approach that addresses the interplay between biological and psychosocial factors. For all patients, routine screening for depression and physical inactivity is important, as these factors are strongly associated with higher pain levels. However, for NHPI patients specifically, addressing metabolic health and BMI is of particular importance, as these variables appear to be key drivers of increased symptom burden in this population. Through adopting a culturally informed, multidimensional care approach that integrates mental health support with targeted physical activities and metabolic care, clinicians may better be able to address the complex manifestations of fibromyalgia and improve outcomes for these underserved populations.

## Figures and Tables

**Table 1.** Clinical Characteristics by Ethnoracial Group:

	Native Hawaiian (n = 55)	Pacific Islander (n = 40)	Asian (n = 35)	White (n = 70)	p-value (raw)	p-value (adjusted)
**BMI**	29.4 ± 5.0 (n=55)	30.1 ± 6.0 (n=40)	24.8 ± 4.5 (n=35)	27.1 ± 5.4 (n=70)	0.0042*	0.0336*
**Age at diagnosis (yrs)**	52.6 ± 15.3 (n=55)	49.7 ± 14.6 (n=40)	47.8 ± 13.9 (n=35)	54.1 ± 16.1 (n=70)	0.125	1.0
**Pain scale**	6.5 ± 2.4 (n=55)	7.0 ± 2.1 (n=40)	5.3 ± 2.2 (n=35)	6.8 ± 2.6 (n=70)	0.063	1.0
**Exercise frequency (days/week)**	2.1 ± 1.3 (n=55)	2.3 ± 1.5 (n=40)	3.5 ± 1.6 (n=35)	3.2 ± 1.7 (n=70)	0.008*	0.064
**Sex - Female (%)**	45 (82%)	32 (80%)	27 (77%)	56 (80%)	0.938	1.0
**Depression (%)**	24 (44%)	20 (50%)	9 (26%)	28 (40%)	0.184	1.0
**Diabetes (%)**	20 (36%)	16 (40%)	4 (11%)	17 (24%)	0.094	1.0
**Hypertension (%)**	22 (40%)	15 (38%)	6 (17%)	22 (31%)	0.136	1.0

**Table 2.** Clinical Characteristics by BMI:

	Normal (BMI ≤25)	Overweight (BMI 26-29)	Obese (BMI ≥30)	p-value (adjusted)
**Age (years)**	48.5 ± 15.2 (n=80)	50.7 ± 14.9 (n=70)	54.9 ± 13.3 (n=90)	0.018*
**Sex Female**	78% (62)	82% (57)	85% (77)	1.0
**Race (selected groups):**				0.20
**Native Hawaiian**	25% (20)	28% (20)	22% (20)	
**Pacific Islander**	15% (12)	16% (11)	18% (16)	
**Asian**	30% (24)	28% (20)	32% (29)	
**White**	30% (24)	28% (20)	28% (25)	
**Pain Scale**	5.5 ± 2.0 (n=80)	6.9 ± 2.3 (n=70)	7.4 ± 2.5 (n=90)	0.009*
**Diabetes (%)**	13% (10)	20% (14)	29% (26)	0.004*
**Hypertension (%)**	16% (13)	23% (16)	31% (28)	0.006*
**Depression (%)**	36% (29)	42% (29)	44% (40)	0.31
**Exercise Frequency**	3.3 ± 1.5 (n=80)	2.9 ± 1.3 (n=70)	2.2 ± 1.2 (n=90)	0.045*

**Table 3.** Clinical Characteristics by Pain Score:

	Below Median Pain (<7)	Above or Equal Median Pain (≥7)	p- value (raw)	p-value (adjusted)
**BMI**	26.8 ± 5.3 (n=104)	29.0 ± 5.7 (n=96)	0.014	0.11
**Age at diagnosis (yrs)**	53.2 ± 16.2 (n=104)	52.4 ± 15.2 (n=96)	0.67	1.0
**Exercise frequency (days/week)**	3.2 ± 1.5 (n=104)	2.5 ± 1.5 (n=96)	0.006*	0.048*
**Sex - Female (%)**	84 (81%)	80 (83%)	0.72	1.0
**Race: Native Hawaiian**	14 (13%)	13 (14%)	0.85	1.0
**Race: Pacific Islander**	17 (16%)	13 (14%)	0.65	1.0
**Race: Asian**	13 (12%)	11 (11%)	0.71	1.0
**Race: White**	60 (58%)	59 (61%)	0.68	1.0
**Depression (%)**	35 (34%)	52 (54%)	0.003*	0.024*
**Diabetes (%)**	17 (16%)	22 (23%)	0.23	1.0
**Hypertension (%)**	23 (22%)	28 (29%)	0.27	1.0

## Abbreviations

NHPI: Native Hawaiian and Pacific Islander
BMI: Body mass index
HPN: Hawaii Pacific Neuroscience
